# Non-integumentary melanosomes can bias reconstructions of the colours of fossil vertebrates

**DOI:** 10.1038/s41467-018-05148-x

**Published:** 2018-07-23

**Authors:** Maria E. McNamara, Jonathan S. Kaye, Michael J. Benton, Patrick J. Orr, Valentina Rossi, Shosuke Ito, Kazumasa Wakamatsu

**Affiliations:** 1 School of Biological, Earth and Environmental Sciences, University College Cork, North Mall, Cork, T23 TK30 Ireland; 2 School of Earth Sciences, University of Bristol, Queen’s Road, Bristol, BS8 1RJ UK; 3 UCD School of Earth Sciences, University College Dublin, Belfield, Dublin, D04D1W8 Ireland; 4 Department of Chemistry, Fujita Health University School of Health Sciences, Toyoake, Aichi 470-1192 Japan

## Abstract

The soft tissues of many fossil vertebrates preserve evidence of melanosomes—micron-scale organelles that inform on integumentary coloration and communication strategies. In extant vertebrates, however, melanosomes also occur in internal tissues. Hence, fossil melanosomes may not derive solely from the integument and its appendages. Here, by analyzing extant and fossil frogs, we show that non-integumentary melanosomes have high fossilization potential, vastly outnumber those from the skin, and potentially dominate the melanosome films preserved in some fossil vertebrates. Our decay experiments show that non-integumentary melanosomes usually remain in situ provided that carcasses are undisturbed. Micron-scale study of fossils, however, demonstrates that non-integumentary melanosomes can redistribute through parts of the body if carcasses are disturbed by currents. Collectively, these data indicate that fossil melanosomes do not always relate to integumentary coloration. Integumentary and non-integumentary melanosomes can be discriminated using melanosome geometry and distribution. This is essential to accurate reconstructions of the integumentary colours of fossil vertebrates.

## Abstract (web summary)

The colour of extinct animals has been inferred from fossilized melanosomes – organelles rich in melanin. Here, the authors show that internal melanosomes that do not contribute to colour are abundant in some extant vertebrates and may redistribute during fossilization, necessitating caution in interpreting fossil colour.

## Introduction

Melanin is a key component of visual signals in animals through its incorporation into integumentary patterning^1^. In vertebrates, melanin occurs in skin and its derivatives as discrete micron-sized membrane-bound organelles termed melanosomes^2^. Physical and chemical evidence of melanin has been used to infer the plumage colours of fossil birds and feathered dinosaurs (reviewed in ref.^3^) and integumentary coloration in fossil marine reptiles^4^ (but see refs.^5–7^). Reconstructions of original skin colour rely on evidence that fossil microstructures are melanosomes (and not, for example, bacteria^5^) and that fossil melanosomes derive solely from the integument. Melanosomes occur, however, in various internal organs in extant vertebrates^8–10^ and thus melanosomes preserved in the body outline of fossils will not always indicate integumentary colour. Given the high recalcitrance of melanin and melanosomes^11^, non-integumentary melanosomes (defined here as those from internal organs and tissues and excluding the eyes) may persist during fossilization, but this remains to be demonstrated; the decay microenvironment within carcasses and the external environment often differ markedly in chemistry^12,13^. If non-integumentary melanosomes are decay-resistant and abundant in vivo, reconstruction of integumentary coloration based on the presence/absence, shape and distribution of fossil melanosomes requires that melanosomes from different tissue sources can be discriminated. Recent studies of colour reconstruction^14,15^ have opted to avoid sampling the torso, in attempts to avoid sampling non-integumentary melanosomes. This does not, however, guarantee that melanosomes from the limbs and tail are integumentary because non-integumentary melanosomes may be redistributed throughout parts of the body during decay. In key biotas such as Jehol, fossils are often partly disarticulated by the action of bottom currents close to the lake floor and can preserve evidence for rupturing of organs and redistribution of their contents throughout the body^16^. Intuitively, such disturbance of exposed carcasses by current activity could also potentially distribute melanosomes from degraded organs inside the body. Whether such redistribution is likely in the absence of physical disturbance (e.g., when organs decompose or the carcass ruptures from gas pressure) is unknown. Resolving these critical issues requires a systematic and comprehensive test of (1) whether non-integumentary melanosomes are abundant and can survive decay and (2) under what taphonomic scenarios are they likely to redistribute.

Here, we address these issues using histology, fluorescence microscopy, scanning electron microscopy (SEM) and alkaline hydroxide peroxide oxidation (AHPO)^11^ of tissues from freshly killed and experimentally decayed extant frogs coupled with data from fossils. Frogs are an ideal test case as melanosomes have been reported in internal organs of extant taxa^8–10^ and fossil examples are known that preserve melanosomes^13^. Our data show that non-integumentary melanosomes are abundant in some extant vertebrates, have a high fossilization potential, and can redistribute throughout the body if disturbed by bottom currents, and must thus be considered in studies of fossil colour based on preserved melanosomes. Non-integumentary and integumentary melanosomes can, however, be discriminated on the basis of their geometry and spatial distributions.

## Results

### Abundance of non-integumentary melanosomes

**Fig. 1:**
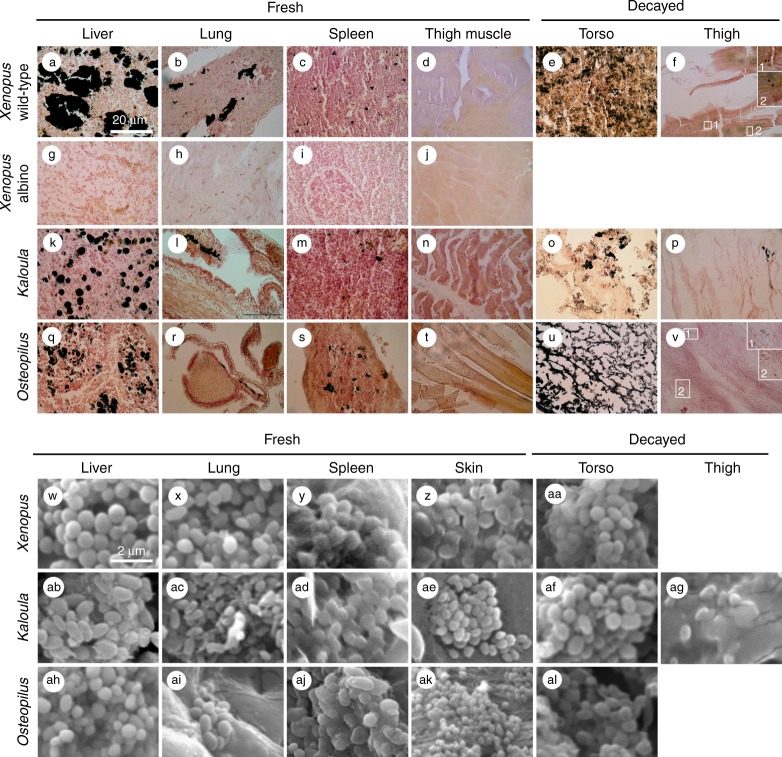
Melanosomes in extant frogs. **a**–**v** Histological sections stained with Fontana-Masson; melanosomes (and aggregates of melanosomes) appear black. Insets in **f** and **v** show isolated melanosomes. **w**–**al** Scanning electron micrographs. Scale bar in **a**, 20 µm, and same scale in **a**–**v**; scale bar in **w**, 2 µm, and same scale in **w**–**al**

Fontana-Masson histological staining is an established technique for the identification of melanin in histological sections^17^: melanosomes are stained black, and other tissue components, hues of pink to yellow (Fig. 1a–v). Fontana-Masson, however, can also stain argentaffin granules, which can occur in liver tissue and in the epithelial lining of the digestive tract. To discriminate between melanosomes and argentaffin granules, we prepared histological sections of tissues from an albino specimen of *Xenopus laevis* (albino animals lack melanosomes but can possess argentaffin granules^18^). The results (Fig. 1g–j) reveal no evidence for argentaffin granules. The regions stained black in the histological sections of the wild-type (melanin-bearing) specimens thus correspond to melanin.

Histological sections and scanning electron microscopy of tissue samples confirm that melanosomes are present in the lung, liver and spleen (but not the thigh; Fig. 1d, n, t) of all three extant taxa (Fig. 1a–c, k–m, q–s). These data are supported by AHPO analyses of *X. laevis* tissue, which confirm the presence of pyrrole-2,3,5-tricarboxylic acid (PTCA), pyrrole-2,3-dicarboxylic acid (PDCA) and pyrrole-2,3,4,5-tetracarboxylic acid (PTeCA) in the liver, lung and spleen (Table 1). These are diagnostic biomarkers for eumelanin^11,19,20^. 4-Amino-3-hydroxyphenylalanine (4-AHP), a specific biomarker for pheomelanin^21^, was also detected after hydroiodic acid hydrolysis. The amounts of 4-AHP are much lower than those of PTCA and PTeCA, indicating that the melanin produced in those internal organs is eumelanic.

**Table 1:** AHPO quantification of melanin markers in extant and fossil frogs

Taxon	Tissue	PTCA (ng/mg)	PDCA (ng/mg)	PTeCA (ng/mg)	PTeCA/PTCA	4-AHP (ng/mg)
*X. laevis*	Liver	128	8.1	60.7	0.47	2.9
	Lung	73.7	5.9	29.2	0.40	4.3
	Spleen	12.8	1.0	17.4	1.36	0.6
*P. pueyoi*	Torso	258	36.9	486	1.88	–

PTCA and PDCA are markers for eumelanin; 4-AHP is a marker for phaeomelanin and PTeCA is a marker for diagenetically altered eumelanin^9^

Volumes of non-integumentary melanosomes were calculated as follows. First, the volume of each organ was measured in the laboratory. The volume of melanosomes in each organ was then estimated using histological sections, whereby the area of tissue occupied by melanin in each section was converted to a volume using the known thickness of the histological sections; see Methods). Volumes of non-integumentary melanosomes are 1.7 cm^3^ for *Xenopus*, 0.36 cm^3^ for *Kaloula* and 0.01 cm^3^ for *Osteopilus* (Table 2); these values correspond to 3.9%, 0.6% and 0.1% of body volume, respectively. For all taxa, melanosomes are most abundant in the liver (84–98% of total melanosomes) and least abundant in the spleen (0.0005–1.45% of total melanosomes; Table 2).

**Table 2:** Abundance and volume of melanosomes in extant *Xenopus*, *Kaloula* and *Osteopilus*

	Area (cm^2^)		Volume of melanosomes in extant frog tissues (cm^3^)				Theoretical volume of melanosomes (cm^3^)	
Taxon	Total body silhouette	Torso silhouette	Lung	Liver	Spleen	Total	Total body silhouette	Torso silhouette
*Xenopus*	50.425	20.235	0.0322	1.6705	0.0008	1.7035	0.126	0.05
*Kaloula*	15.201	11.215	0.005	0.35	0.0002	0.3552	0.038	0.028
*Osteopilus*	11.302	3.779	0.0001	0.0104	0.0002	0.0116	0.028	0.009

Models of integumentary colour in fossils should consider the potential of contributions from non-integumentary melanosomes if the latter are abundant in extant vertebrates. The abundance of non-integumentary melanosomes was assessed as follows. The layer of non-integumentary melanosomes in the Libros frogs has a mean thickness of ca. 25 µm (measured in SEM from polished vertical sections)^12^. The volume of melanosomes required to produce a layer of this thickness over the body outline is 0.126 cm^3^ for *Xenopus*, 0.038 cm^3^ for *Kaloula* and 0.028 cm^3^ for *Osteopilus* (Table 2). Remarkably, *Xenopus* and *Kaloula* therefore possess sufficient non-integumentary melanosomes to define a layer over the entire body that is 338 μm thick and 234 μm thick, respectively, i.e., far in excess of the thickness of the layer in the fossils. *Osteopilus* has sufficient non-integumentary melanosomes to define a 25-μm-thick layer over the torso, but not the torso plus the limbs. In extant frogs, integumentary melanosomes typically form patchy layers <10 μm thick (melanophores (the dominant source of integumentary melanosomes) often occur in clusters)^9^. Thus, in the fossil frogs, and in extant *Xenopus* and *Kaloula*, non-integumentary melanosomes vastly outnumber those from the skin and should dominate the preserved melanosomes.

### The fate of non-integumentary melanosomes during decay

**Fig. 2:**
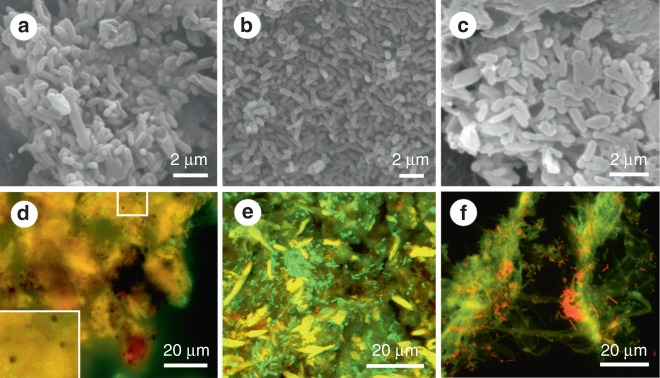
Decayed tissues of extant frogs. **a**–**c** Scanning electron micrographs of rod-shaped decay bacteria within internal tissues of the torso in *Kaloula* (**a**), *Xenopus* (**b**) and *Osteopilus* (**c**). **d**–**f** Fluorescent micrographs of melanosomes (**d**) and rod-shaped bacteria (**e**) in the torso, and of rod-shaped bacteria in the thigh (**f**). Melanosomes appear black in fluorescent images. Bacteria appear green and red. Scale bars, 2 μm (**a**–**c**), 20 μm (**d**–**f**)

Scanning electron micrographs of the tissues of the experimentally degraded frogs reveal abundant rod-shaped and spherical to oblate microstructures (Figs. 1aa, af, ag, al and 2a–c). The rod-shaped microstructures are interpreted as decay bacteria as no rod-shaped structures were identified in the tissues in untreated specimens. The spherical to oblate microstructures, however, are not definitively coccoid bacteria, as they and melanosomes cannot be discriminated in scanning electron micrographs. In fluorescence images of the degraded tissues, rod-shaped microstructures stain green (Fig. 2d–f) and can be identified as bacteria (in stationary growth phase) on the basis of their fluorescence response^22^, rod-shaped morphology, size (1.5–2.5 µm long), spatial association with identical rod-shaped microstructures that stain orange-red (representing bacteria in active growth phase), and location within regions of filamentous material, presumably bacterial extracellular polymeric saccharides. The spherical, micron-sized structures in the fluorescence images comparable to those visible under SEM quench fluorescence and appear dark (Fig. 2d–f), as with melanosomes in extant animals^23–25^. This plus the melanin-rich nature of our tissue samples and the histological evidence for melanosomes in the degraded tissues from the torso (Fig. 1e, o, u) and thigh (Fig. 1f, p, v), the dark microstructures in the fluorescence images can be most parsimoniously interpreted as melanosomes. Melanin can fluoresce when strongly oxidized using hydrogen peroxide^25,26^, but that procedure was not used in this study.

Our laboratory experiments thus confirm that non-integumentary melanosomes survive the decay process; as with melanosomes from the skin, they show no visible morphological alteration during decay. Our results also show that melanosomes usually remain dispersed through, but inside, in the torso during early decay. After 12 weeks, non-integumentary melanosomes are abundant in degraded tissue from the torso (Fig. 1e, o, u, aa, af, al). They also occur, albeit rarely, in the thigh region of all three taxa (Fig. 1f, p, v, aa, ag). Given that our experiments did not involve agitation or other disturbance of carcasses, our data suggest that substantial redistribution of melanosomes in fossils is unlikely where carcasses are deposited prior to extensive decay and not subjected to disturbance while resting on the sediment surface, e.g., by bottom currents. More prolonged pre-depositional decay and/or post-depositional disturbance of carcasses, however, is likely to result in more extensive redistribution of melanosomes inside the body. This hypothesis can be tested using fossil material (see below).

### Non-integumentary melanosomes in fossil amphibians

**Fig. 3:**
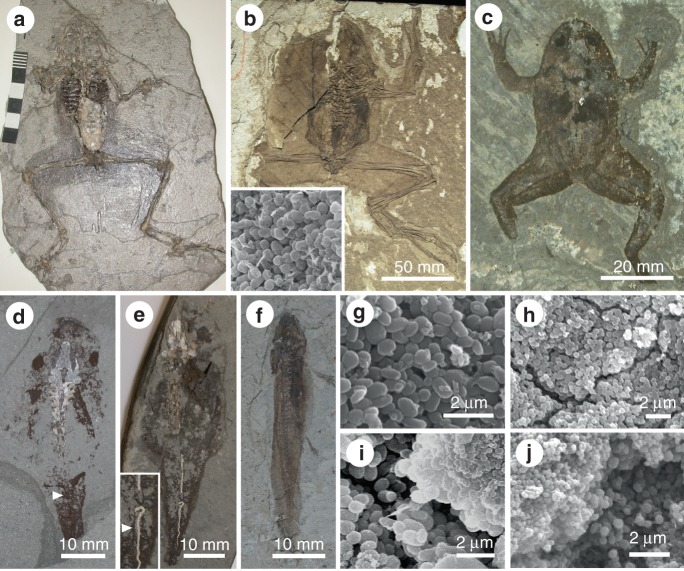
Fossil amphibian soft tissues. **a**–**c** Frogs from Libros (Miocene, Spain) (**a** MNCN 63776, Museu Nacional de Ciencias Naturales, Madrid), Bechlejovice (Oligocene, Czech Republic) (**b** NMP 39449, National Museum Prague), and Messel (Eocene, Germany) (**c** SMF-ME-00978, Forschungsinstitut und Naturmuseum Senckenberg, Frankfurt). Torsos show dark patches of non-integumentary melanosomes. Inset in **b** shows melanosomes from the torso. **d**, **e** Tadpoles from Libros. Lungs are dark patches in the torso (**d** MNCN 63848); the notochord, two closely spaced parallel lines (**d**, **e** NHM 49999, Natural History Museum, London, UK). **f** Salamander from Daohuguo (Middle Jurassic; CNU-V-1264, Capital Normal University, Beijing, China). Torso shows conspicuous dark patches. **g**–**j** Scanning electron micrographs of melanosomes in frogs (**g**–**i**) and tadpoles (**j**) from Libros. **g** Non-integumentary melanosomes. **h** Integumentary melanosomes. **i**, **j** Size-specific layers of melanosomes. **i** Integumentary melanosomes (top right) overlying non-integumentary melanosomes (lower left). **j** Size-specific layers of non-integumentary melanosomes, reflecting vertical superposition of different internal tissues in vivo. Scale bars, 60 mm (**a**), 50 mm (**b**), 20 mm (**c**), 10 mm (**d**, **f**), 2 µm (**g**–**j**)

Previous study of the soft tissues of frogs from the Late Miocene Libros biota revealed the presence of carbonaceous microbodies (Fig. 3g–i)^12^. The microbodies were originally interpreted as fossil bacteria^12^, but reinterpreted as fossil melanosomes based on geochemical data^13^. This interpretation is further supported herein by our AHPO analysis, which confirms the presence of PTCA and PTeCA (a marker for diagenetically altered eumelanin^11^) in the fossil soft tissues (Table 1).

**Fig. 4:**
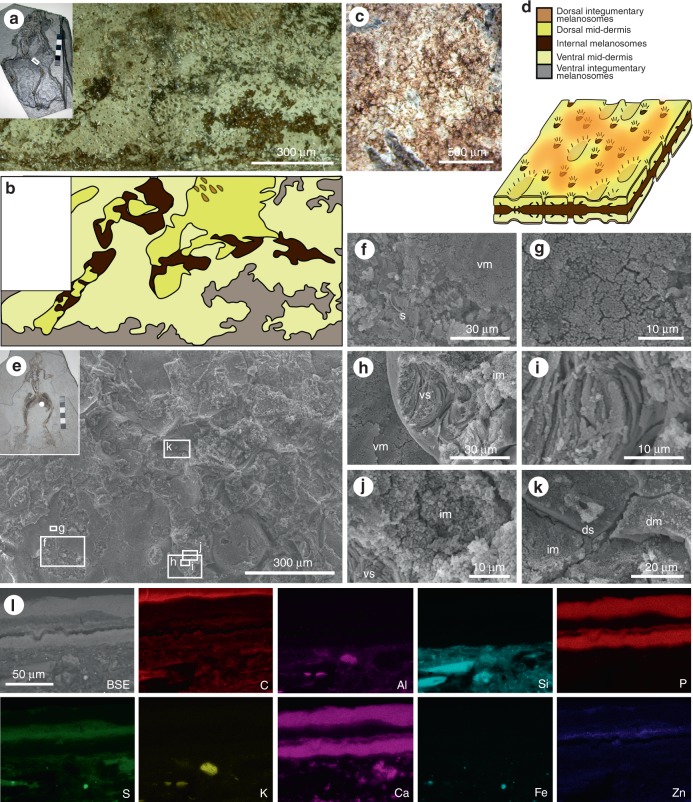
Layered soft tissues in the hindlimbs of fossil frogs from Libros. **a**–**b** MNCN 63775. Light micrograph of area indicated in inset, showing a plan view of the soft tissues in the thigh. **b** Interpretative drawing of soft tissue layers present, based on light microscopy. **c** Patterning in the integumentary melanosome layer. **d** Three-dimensional schematic illustration of the preserved 3D structure of the layered soft tissues in the Libros frogs, based on data presented in ref.^12^ and this figure. The terms ‘dorsal’ and ‘ventral’ are for illustrative purposes only. **e**–**l** MNCN 63798. **e** Scanning electron micrograph of soft tissues from the region indicated in the inset. **f**–**k** Details of regions indicated in **e**. **f** Sediment (s) underlying the ventral melanosome layer (vm). **g** Detail of ventral melanosomes. **h** Ventral layer of phosphatized skin (vs) overlain by non-integumentary melanosomes (im) and underlain by ventral melanosomes. **i** Detail of collagen fibres of phosphatized skin. **j** Detail of non-integumentary melanosomes. **k** Dorsal layer of phosphatized skin (ds) overlain by dorsal melanosome layer (dm). **l** Elemental maps of polished sections through the soft tissues from the thigh. Melanosome layers are defined by C, S and Zn. SE: secondary electron micrograph of region analysed, dm: dorsal melanosome layer, s: sediment, vm: ventral melanosome layer, vs: ventral skin layer. Scale bars, 300 μm (**a**), and same scale in **b**, 500 μm (**c**),  300 µm (**e**), 30 µm (**f**, **h**), 10 µm (**g**, **i**, **j**), 20 µm (**k**), 50 μm (**l**)

The melanosomes occur as layers in association with phosphatized soft tissues interpreted as the mid-dermal Eberth–Katschenko (E–K) layer. This interpretation is discussed in detail in ref.^12^; in brief, the fossil layer is identical to the E–K layer of extant frogs in terms of its anatomical distribution, thickness, collagen-rich composition, the geometry of its upper and lower surfaces, and the presence of perforations identical in diameter and geometry to the centrifugal pillars of the dermis. Selective phosphatization of this tissue in the fossils reflects its phosphate-rich composition in vivo. This phosphatized tissue layer is both under- and overlain by layers of melanosomes (‘internal’ and ‘external’, respectively). These soft tissue layers have different characteristics in hand specimen (Fig. 4): the outer melanosome layer is light brown in colour, 2–5 µm thick, can be patchy or continuous and can preserve patterning (Fig. 4c); the phosphatized mid-dermis is white to cream in colour, 15–30 µm thick, and continuous; and the inner melanosome layer is dark brown in colour, 15–200 µm thick, and continuous. Both the outer melanosome layer and the phosphatized mid-dermal layer are present on dorsal and ventral sides of the specimen.

Our results demonstrate that the melanosomes in different layers are from different tissues. Melanosomes in the outer layer are 0.35–0.5 µm spheres; the inner layer includes sub-layers comprising 0.9–1.1 µm ovoids and ca. 0.5 µm spheres, respectively (Fig. 3i)^12,13^. The outer melanosome layer derives from the upper dermis and epidermis, and the thicker inner layer, from internal tissues.

Other amphibians from Libros and other localities^27,28^ also exhibit discrete layers of melanosomes. Thin patchy layers over the body outline represent integumentary melanosomes; non-integumentary melanosomes form a thicker layer that is usually confined to the torso (Fig. 3a–f), where they can define the positions of internal organs^12,27–29^. Critically, 93% of the 73 Libros frogs studied possess non-integumentary melanosomes (see Supplementary Data 1) and in 25%, the latter extend from the torso into the limbs^29^ (Fig. 4). Thus, it cannot be assumed a priori that melanosomes from the body outlines of fossil vertebrates represent only integumentary melanosomes. A mechanism to discriminate integumentary and non-integumentary melanosomes is required.

### Tissue-specific melanosome geometries

Our results show that melanosomes vary in geometry among body tissues within individual taxa (Table 3); except for the liver, lung and spleen of *Osteopilus* (which have similar geometries), these differences are statistically significant (Table 4). Critically, differences in the sizes of melanosomes from the skin and the liver (the primary source of melanosomes) are statistically significant for all three taxa (Table 4). We predict that collapse of soft tissues during decay would generate diagnostic spatial distributions of melanosomes, typically a thick layer of melanosomes sandwiched between thin layers of melanosomes of different geometry; where melanosomes have not redistributed, limbs should show a thin layer of melanosomes of uniform size. Fossil frogs from Libros exhibit the predicted vertical partitioning of melanosomes (Fig. 3g–i): non-integumentary and integumentary melanosomes differ significantly in geometry (Student’s *t*-test *p* = 3.42e^−13^ (length); *p* = 7.96e^−8^ (aspect ratio)); non-integumentary melanosomes from adjacent layers differ in geometry (*p* = 1.67e^−17^ (length); *p* = 1.28e^−8^ (aspect ratio)).

**Table 3:** Geometry of melanosomes in extant *Xenopus*, *Kaloula* and *Osteopilus*

Taxon	Tissue	Melanosome length (µm)	Melanosome width (µm)	Melanosome aspect ratio
*Xenopus*	Lung	1.038 ± 0.149	0.671 ± 0.113	1.571 ± 0.258
	Liver	0.747 ± 0.136	0.669 ± 0.113	1.121 ± 0.114
	Spleen	0.887 ± 0.129	0.625 ± 0.078	1.422 ± 0.172
	Skin	0.969 ± 0.163	0.643 ± 0.099	1.539 ± 0.266
*Kaloula*	Lung	0.966 ± 0.117	0.49 ± 0.0436	2.041 ± 0.434
	Liver	1.042 ± 0.103	0.711 ± 0.093	1.481 ± 0.174
	Spleen	0.76 ± 0.182	0.68 ± 0.124	1.17 ± 0.247
	Skin	0.53 ± 0.081	0.404 ± 0.051	1.323 ± 0.22
*Osteopilus*	Lung	0.827 ± 0.124	0.545 ± 0.069	1.532 ± 0.27
	Liver	0.9 ± 0.115	0.614 ± 0.113	1.492 ± 0.331
	Spleen	0.942 ± 0.173	0.65 ± 0.102	1.449 ± 0.28
	Skin	0.418 ± 0.049	0.332 ± 0.03	1.272 ± 0.226

**Table 4:** Student’s *t*-test analysis of differences in melanosome geometry between each pair of tissue types listed in Table 3

***Xenopus*** **melanosome length**				***Xenopus*** **melanosome aspect ratio**			
	Lung				Lung		
Liver	*1.57e* ^***−****7*^	Liver		Liver	*1.38e* ^***−****7*^	Liver	
Spleen	*0.001*	*0.001*	Spleen	Spleen	*0.0394*	*2.1e* ^***−****7*^	Spleen
Skin	*0.005*	*1.63e* ^***−5***^	0.265	Skin	0.8528	*2.03e* ^***−****7*^	*0.0146*
***Kaloula*** **melanosome length**				***Kaloula*** **melanosome aspect ratio**			
	Lung				Lung		
Liver	*0.034*	Liver		Liver	*1.5e* ^***−****5*^	Liver	
Spleen	*1.52e* ^***−****4*^	*1.02e* ^***−****7*^	spleen	Spleen	*6.28e* ^***−****8*^	*2.72e* ^***−****4*^	Spleen
Skin	*2.58e* ^*−15*^	*4.26e* ^*−19*^	*1.21e* ^*−11*^	Skin	*3.63e* ^***−****7*^	*0.0168*	0.088
***Osteopilus*** **melanosome length**				***Osteopilus*** **melanosome aspect ratio**			
	Lung				Lung		
Liver	0.111	Liver		Liver	0.671	Liver	
Spleen	0.256	0.672	Spleen	Spleen	0.53	0.85	Spleen
Skin	*4.55e* ^*−13*^	*3.57e* ^*−12*^	*1.01e* ^*−11*^	Skin	0.926	*0.02*	*0.037*

Tests returning a significant result are indicated in italicized font

## Discussion

This study highlights a potential risk in the use of fossil melanosomes to infer original coloration in ancient animals. Melanosomes preserved within the body outline of a fossil animal may not have imparted integumentary coloration.

Contrary to suggestions that melanosomes decay rapidly^5^, our experiments confirm that vertebrate melanosomes routinely survive decay. Analysis of modern frogs shows that non-integumentary melanosomes are highly abundant and most remain in situ during early decay provided carcasses are not disturbed. Many fossils, especially those deposited rapidly after death in a quiescent environment, and especially those buried soon after death, may thus be expected to retain original distributions of non-integumentary melanosomes. Our fossil analysis demonstrates, however, that under certain circumstances, non-integumentary melanosomes can redistribute from the torso into the limbs; many frogs from Libros show evidence of disturbance by weak currents during residence on the lake floor, viz. disarticulation of small distal limb bones and association of fossils with plant fragments^29^. Redistribution should thus be anticipated in contexts where the biostratinomy of the fossils or the sedimentological context indicates current activity.

**Fig. 5:**
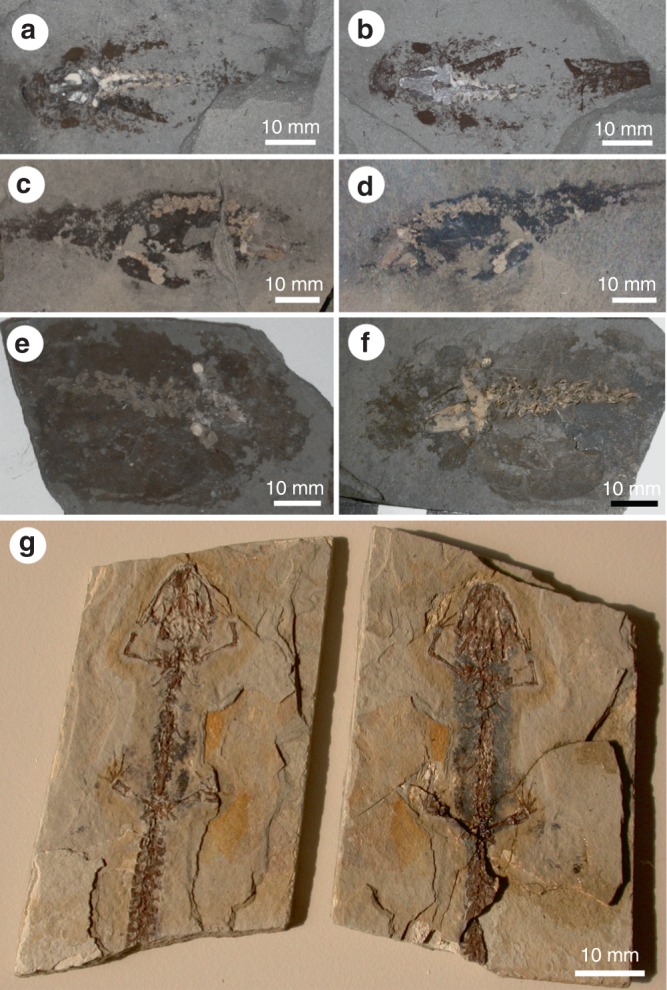
Distribution of soft tissues in part and counterpart of fossil amphibian specimens. In **a**–**f** the plane of splitting passes medially through the soft tissues, thus exposing non-integumentary melanosomes at the surface and producing near-identical distributions of soft tissues in part and counterpart. In **g**, the dissimilar distribution of soft tissues in part and counterpart demonstrates that the plane of splitting is not precisely medial within the soft tissues; non-integumentary melanosomes may still be exposed at the surface. **a** MNCN 63781. **b** MNCN 63848. **c** MNCN 63793. **d** MNCN 63864. **e** IPS 16465, Institut de Paleontología de Sabadell Miquel Crusafont, Sabadell, Barcelona, Spain. **f** MGB-33179a, Museu de Geologia de Barcelona, Barcelona, Spain. **g** MPV-1934-RMa, Museu de Paleontología de Valencia, Valencia, Spain. **a**–**f** are larval *P. pueyoi* from Libros; **g** is *Chelotriton* from the Middle Miocene of Rubielos de Mora, Spain. Scale bars, 10 mm

Even if they remain in situ, melanosomes preserved in the torso will derive from both the internal organs and the skin and thus cannot be assumed to reflect integumentary pigmentation. Compounding this, the plane of splitting typically passes through preserved soft tissues (as evidenced by similar distributions of soft tissues on both part and counterpart of a specimen; Fig. 5a–f), thus exposing non-integumentary melanosomes on the surface of a fossil. Importantly, preservation of non-integumentary melanosomes does not necessarily preclude preservation of original integumentary colour patterning (Fig. 4c), especially where melanosomes have not redistributed and in body regions where redistribution is unlikely. The latter includes feathers that do not overlap the torso, in particular where melanosomes are localized to the barbules and are embedded in an organic matrix, i.e., the degraded remains of the feather keratin. Studies of fossil melanin should consider the possibility that variations in tone in fossil soft tissues comprising melanosomes may reflect the positions of internal organs where melanosomes have remained in situ, or the presence of layers of melanosomes redistributed from internal organs, not integumentary patterning.

Our results show that melanosomes from the internal organs and skin in extant frogs can, in some instances, be discriminated based on geometry. Sympathetic to this, the fossils studied show vertical partitioning of melanosomes into size-specific layers. These observations form a basis for assessing the suitability of fossil specimens for studies of integumentary coloration. Ideal specimens are those that retain original spatial distributions of melanosomes, evident in SEM images as vertical and lateral separation of melanosomes of different geometries within the preserved soft tissues. Such specimens will characteristically show definition of internal organs by dark tones in hand specimen and offer the best opportunity to prevent erroneous sampling of non-integumentary melanosomes. Layering of melanosomes has also been identified in the eyespots of fossil animals and interpreted as a taxonomic signal^30^. A single visually uniform layer of melanosomes (potentially with mixing of melanosomes of different size and geometry) in fossil specimens, however, may be original or result from melanosome dispersal.

**Fig. 6:**
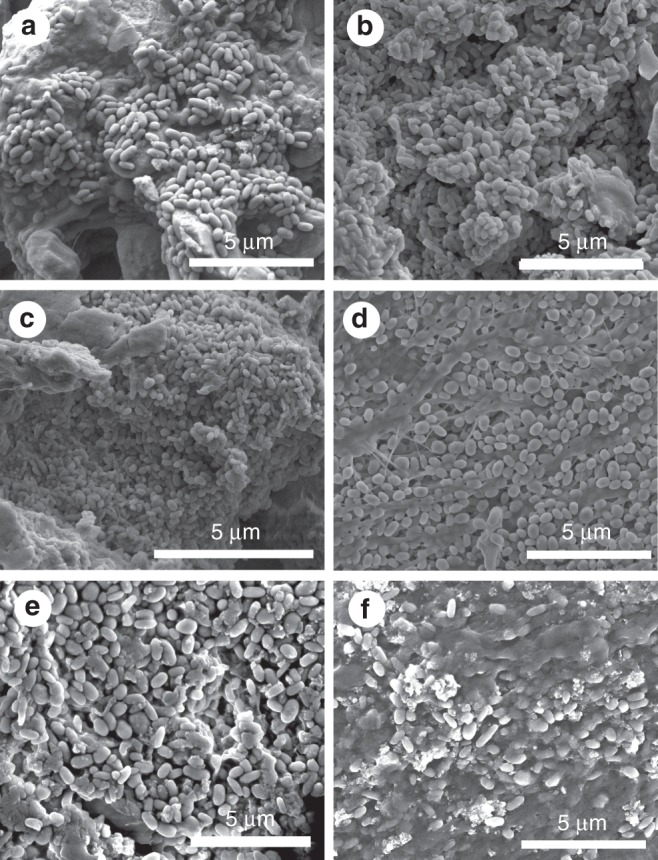
Melanosomes in extant reptiles. Scanning electron micrographs of extant black iguana (*Ctenosaura similis* (Squamata: Corytophanidae), **a**–**c**) and the common basilisk (*Basiliscus basiliscus* (Squamata: Iguanidae), **d**–**f**). **a**, **d** Connective tissue. **b**, **e** Liver. **c** Kidney. **f** Spleen. Scale bars, 5 μm

Diverse fossil vertebrate taxa preserve visual evidence for non-integumentary melanosomes and thus our results have broader applications within vertebrates, including taxa that possess feathers or hair. Examples include the conspicuous dark patch in the anterior torso of the ichythyosaur *Stenopterygius quadriscissus*^31^ and dark patches in the torso of the bat *Palaeochiropteryx tupadiodon*^32^. Numerous examples in fossils from the Jehol biota include the dark patches in the torso of *Sinosauropteryx prima*^33^, *Sinornithosaurus*^34^ and *Epidexipteryx*^35^. Preliminary analyses of extant reptiles confirm that melanosomes are abundant in various internal tissues (Fig. 6). Future studies of diverse extant vertebrates will characterize the preservation potential, abundance and geometry of non-integumentary melanosomes, and will test the feasibility of other mechanisms to identify the source of melanosomes, e.g., the chemistry of the melanosomes and their embedding matrix. Discriminating between the melanosomes from different sources is essential to accurate reconstruction of melanin-based pigmentation in fossil vertebrates.

## Methods

### Decay experiments

The morphological decay of frogs has been studied in detail previously^36^. Our laboratory experiments had two objectives: (1) to assess whether non-integumentary melanosomes survive early decay, and (2) to assess whether these melanosomes redistribute within a carcass during early decay. We conducted a series of pilot experiments using extant frogs to identify the most appropriate conditions for our experiments; the goal was not to attempt to replicate the full complexity of natural settings^37^ but to identify conditions in which carcasses would remain intact during decay, allowing the decay of melanosomes within the body cavity to be tracked. Adult specimens of the African-clawed frog, *Xenopus laevis* (Anura: Pipidae), the Asian-painted frog, *Kaloula pulchra* (Anura: Microhylidae), and the Cuban tree frog, *Osteopilus septentrionalis* (Anura: Hylidae) were euthanased via immersion in water with added 3 g/l tricaine methane sulphonate. The first pilot experiment used natural lake water plus an inoculum of lake sediment and was carried out at 25 °C. This experiment was unsuccessful as all carcasses bloated rapidly due to high rates of gas production by decay bacteria and ruptured within 96 h, releasing internal tissues (and their melanosomes) into the decay medium. The second pilot experiment used distilled water with no sediment and was carried out at 15 °C, but all carcasses ruptured within 1 week. The third pilot experiment involved freezing freshly killed carcasses at −80 °C for 24 h in order to inhibit the growth of (and rate of gas production by) decay bacteria and thus prevent bloating; carcasses were then decayed as per the second phase of pilot experiments. Carcasses in this experiment did not rupture. Dissection of specimens after 3 days revealed that all major organs showed a similar fidelity to those in specimens that had not been frozen; there is no evidence that freezing affected the rate of tissue degradation and/or the redistribution of non-integumentary melanosomes.

The final experiment used five specimens of *Xenopus* (four wild type, one albino) and two specimens each of *Kaloula* and *Osteopilus*. Frog carcasses were frozen at −80 °C for 48 h, placed in individual glass vessels containing 650 ml of distilled water and degraded at 15± 1 °C for 12 weeks in a Memmert IPS260 constant temperature incubator.

Reptiles for scanning electron microscopy (see below) were supplied as carcasses.

### Ethics

The authors have complied with all relevant ethical regulations. Euthanasia of frogs was approved by the University of Bristol Animal Ethics Committee via authorization UIN/13/017 and carried out by the University of Bristol Animal Services Unit.

### Histology

The abundance of melanin in internal organs was assessed using histological sections. Small (ca. 5 mm^3^) samples of liver, lung and spleen were dissected from freshly killed frog specimens and the volume of each organ (and that of the entire frog) calculated by immersion in water. Samples of degraded tissue were also removed from within the abdominal body cavity of degraded specimens. Tissue samples were fixed in 10% neutral buffered formalin overnight and dehydrated in the following ethanol: water mixtures: distilled water (3 × 20 min), 30% ethanol (20 min), 50% ethanol (20 min), 70% ethanol (20 min), 90% ethanol (20 min) and 100% ethanol (2 × 20 min). Dehydrated samples were immersed in histolene-clearing agent overnight and embedded in paraffin wax. Histological sections (9 μm thick) were stained using Fontana-Masson (FM) using standard protocols^17^.

### Image analysis

In histological sections stained using Fontana-Masson, melanosomes appear black. In order to assess the abundance of melanosomes in each organ, the area of tissue occupied by melanosomes was calculated by analyzing digital images of histological sections using the Threshold Colour function in ImageJ (www.imagej.nih.org). Ten images were analyzed for each tissue type in each taxon. These data were used to estimate the volume of melanosomes in each organ based on the measured organ volume (see above) and thickness of histological sections (9 µm).

The mean thickness of the internal melanosome layer in fossil frogs from Libros is 25 μm^12^. The volume of a 25-µm-thick layer corresponding to the torso, and entire body, respectively, was calculated for each extant taxon.

### Fluorescence microscopy

Acridine orange staining is an established technique for discrimination of bacterial cells and eukaryotic tissue in histological sections^17,38^. Eukaryotic cells fluoresce green and prokaryotic cells fluoresce red-orange^19,38^. The staining response of bacteria, however, varies with physiology: active bacteria fluoresce red, and those in the stationary growth phase fluoresce green^19,39^. Sections of stained decayed tissue were stained using Acridine Orange using standard protocols^17,38^ and analyzed using an Olympus BX53 upright fluorescence microscope (×100 objective, N.A. 1.4) with filter cube sets for green (Exciter filter 470–495, DM505, Barrier filter 510–550 nm) and blue images (Exciter filter 540–550, DM570, Barrier filter 576–625 nm).

### Scanning electron microscopy (SEM)

Samples of fresh and decayed tissue were prepared using standard protocols^40^ and examined using a Hitachi S-3500N variable pressure SEM at an accelerating voltage of 15 kV. Long and short axes were measured for melanosomes from the liver, lung, spleen and skin in the extant frogs, and from melanosomes in the two layers in the fossils.

### Alkaline hydroxide peroxide oxidation (AHPO)

Alkaline hydrogen peroxide oxidation is a unique chemical assay for melanin that produces diagnostic chemical markers (e.g., pyrrole-2,3,5-tricarboxylic acid (PTCA), pyrrole-2,3-dicarboxylic acid (PDCA) and pyrrole-2,3,4,5-tetracarboxylic acid (PTeCA), which are derived from the dihydroxyindole parent subunit of the melanin molecule) that allow identification and quantification of melanin in modern and fossil materials^11,19^. Freeze-dried samples of liver, lung and spleen of *X. laevis* (9–17 mg) were homogenized in water with a Ten-Broeck homogenizer at a concentration of 10 mg/ml and 200 µl aliquots were dried in a dessicator and  subjected to acid hydrolysis with 6 M HCl (0.5 ml) at 110 °C for 16 h^40^. This acid hydrolysis removes proteins and small molecules that might interfere with the assay and thus increases specificity of the biomarkers^40^. The resulting insoluble materials including melanin were collected by centrifugation and washed once with water as described^40^. Then the residues were subjected to alkaline hydrogen peroxide oxidation (AHPO)^38^. Aliquots (100 µl) of *X. laevis* suspensions were also subjected to hydroiodic acid hydrolysis to analyze 4-amino-3-hydroxyphenylalanine (4-AHP), a specific biomarker for phaeomelanin^39^. A sample of soft tissues from a fossil frog from the Late Miocene Libros biota (MNCN 63776) was finely ground with a mortar and pestle and weighed. The powder was subjected to acid hydrolysis and then AHPO as above.

### Data availability

The SEM data that support the findings of this study can be downloaded from the CORA repository (www.cora.ucc) at http://hdl.handle.net/10468/6452.

## Electronic supplementary material

Description of Additional Supplementary Files

Supplementary Data 1

## References

[1] McGraw, K. in *Bird Coloration: Function and Evolution* (eds Hill, G. & McGraw, K.) 243–294 (Harvard University Press, Cambridge, MA, 2006).

[2] Marks M, Seabra M (2001). The melanosome: membrane dynamics in black and white. Nat. Rev. Mol. Cell Biol..

[3] McNamara ME (2013). The taphonomy of colour in fossil insects and feathers. Palaeontology.

[4] Lindgren J (2014). Skin pigmentation provides evidence of convergent melanism in extinct marine reptiles. Nature.

[5] Moyer A (2014). Melanosomes or microbes: testing an alternative hypothesis for the origin of microbodies in fossil feathers. Sci. Rep..

[6] Lindgren J (2015). Interpreting melanin-based coloration through deep time: a critical review. Proc. R. Soc. B.

[7] Schweitzer MH, Lindgren J, Moyer AE (2015). Melanosomes and ancient coloration re-examined: a response to Vinther 2015. Bioessays.

[8] Sichel G, Scalia M, Mondio F, Corsaro C (1997). The amphibian Kupffer cells build and demolish melanosomes: an ultrastructural point of view. Pigment Cell Res..

[9] Aughey E, Frye F (2001). Comparative Veterinary Histology.

[10] Scalia M (2004). The spleen pigment cells in some amphibians. Pigment Cell Res..

[11] Glass K (2012). Direct chemical evidence for eumelanin pigment from the Jurassic period. Proc. Natl Acad. Sci. USA.

[12] McNamara ME (2009). Soft tissue preservation in Miocene frogs from Libros (Spain): insights into the genesis of decay microenvironments. Palaios.

[13] McNamara ME, van Dongen BE, Lockyer NP, Bull ID, Orr PJ (2016). Fossilization of melanosomes via sulfurization. Palaeontology.

[14] Hu D (2018). A bony-crested Jurassic dinosaur with evidence of iridescent plumage highlights complexity in early paravian evolution. Nat. Commun..

[15] Li Q (2014). Melanosome evolution indicates a key physiological shift within feathered dinosaurs. Nature.

[16] O’Connor J (2018). First report of gastroliths in the Early Cretaceous bird. Jeholornis. Cretac. Res..

[17] Sheehan D, Hrapchak B (1987). Theory and Practice of Histotechnology.

[18] Gregg RV (1966). The gastric argentaffin cell population of the rat. J. Morphol..

[19] Ito S (2011). Usefulness of alkaline hydrogen peroxide oxidation to analyze eumelanin and pheomelanin in various tissue samples: application to chemical analysis of human hair melanins. Pigment Cell Melanoma Res..

[20] Ito, S. et al. Acid hydrolysis reveals a low but constant level of pheomelanin in human black to brown hair. *Pigment Cell Melanoma Res.* **31**, 393-403 (2018). 10.1111/pcmr.12673 29171182

[21] Wakamatsu K, Ito S, Rees JL (2002). Usefulness of 4-amino-3-hydroxyphenylalanine as a specific marker of pheomelanin. Pigment Cell Res..

[22] McFeters G, Singh A, Byun S, Callis P, Williams S (1991). Acridine orange staining reaction as an index of physiological activity in *Escherichia coli*. J. Microbiol. Methods.

[23] Eldar A, Bejerano Y, Livoff A, Horovitcz A, Bercovier H (1995). Experimental streptococcal meningo-encephalitis in cultured fish. Vet. Microbiol..

[24] Ancans J (2001). Melanosomal pH controls rate of melanogenesis, eumelanin/phaeomelanin ratio and melanosomes maturation in melanocytes and melanoma cells. Exp. Cell Res..

[25] Jarrett I, Spearman RJ (1957). The keratin defect and hair-cycle of a new mutant (matted) in the house-mouse. J. Embryol. Exp. Morphol..

[26] Fuller B, Spaulding D, Smith DR (2001). Regulation of the catalytic activity of preexisting tyrosinase in black and Caucasian human melanocyte cell cultures. Exp. Cell Res..

[27] McNamara ME (2010). Exceptionally preserved tadpoles from the Miocene of Libros, Spain: ecomorphological reconstruction and the impact of ontogeny upon taphonomy. Lethaia.

[28] Špinar, Z. *Tertiary Frogs from Central Europe* (Academia, Prague, 1972).

[29] McNamara ME (2012). What controls the taphonomy of exceptionally preserved taxa – environment or biology? A case study using exceptionally preserved frogs from the Miocene Libros Konservat-Lagerstätte, Spain. Palaios.

[30] Clements T (2016). Eyes of *Tullimonstrum gregarium* reveal a vertebrate affinity. Nature.

[31] Marek R (2015). Fossil focus: ichthyosaurs. Palaeontol. Online.

[32] Schaal S, Ziegler W (1992). Messel: An Insight into the History of Life and of the Earth.

[33] Chen P, Dong Z, Zhen S (1998). An exceptionally well-preserved theropod dinosaur from the Yixian Formation of China. Nature.

[34] Xu X, Wang XL, Wu XC (1999). A dromaeosaurid dinosaur with a filamentous integument from the Yixian Formation of China. Nature.

[35] Zhang F, Zhou Z, Xu X, Wang X, Sullivan C (2008). A bizarre Jurassic maniraptoran from China with elongate ribbon-like feathers. Nature.

[36] Wuttke M (1983). Aktuopaläontologische studien über den Zerfall von Wirbeltieren. Teil 1: Anura. Senck. Lethaia.

[37] Purnell (2018). Experimental analysis of soft-tissue fossilization – opening the black box. Palaeontology.

[38] McCarthy L, Senne J (1980). Evaluation of acridine orange stain for detection of microorganisms in blood cultures. J. Clin. Microbiol..

[39] Hayat M (1993). Stains and Cytochemical Methods.

[40] McNamara (2006). High fidelity preservation of bone marrow in c. 10 million year old amphibians. Geology.

