# Supplementary material for: Non-integumentary melanosomes can bias reconstructions of the colours of fossil vertebrates
Source: Nat Commun. 2018 Jul 23;9:2878. doi: 10.1038/s41467-018-05148-x (PMC6056411; doi:10.1038/s41467-018-05148-x)
Supplement: Supplementary file 1 — Description of Additional Supplementary Files [file 41467_2018_5148_MOESM1_ESM.pdf]

Description of Additional Supplementary Files

File Name: Supplementary Data 1
Description: List of specimens of adult frogs from the Libros biota with (y) and without (n) non-integumentary melanosomes. Dash indicates the presence / absence of non-integumentary melanosomes could not be determined for that specimen, usually because the torso is obscured by sediment.
